# Research on the Process of Cutting Nonwoven Materials Using Surgical Gauze as an Example

**DOI:** 10.3390/ma18133049

**Published:** 2025-06-27

**Authors:** Marcin Zastempowski, Andrzej Bochat, Maciej Janiec

**Affiliations:** 1 Faculty of Mechanical Engineering, Bydgoszcz University of Science and Technology, Al. Prof. S. Kaliskiego 7, 85-796 Bydgoszcz, Poland; andrzej.bochat@pbs.edu.pl; 2 SORIMEX sp. z o.o., ul. Równinna 25, 87-100 Toruń, Poland; janiec56@wp.pl

**Keywords:** surgical gauze, cutting, drum cutting unit, unit cutting resistance, unit cutting work

## Abstract

The aim of this study was to examine the functional characteristics of the process of cutting surgical gauze with a drum cutting unit. For this purpose, the authors designed and constructed a test stand on which experimental tests were conducted. As part of this study, the results of the experimental tests are presented, which were conducted for three selected thicknesses of surgical gauze samples, four selected angles of feeding of the material to be cut and nine selected cutting speeds. In order to determine cutting resistance, the specific cutting resistance was used, and the energy consumption was estimated using the specific cutting work related to the cutting surface of the surgical gauze. The conducted experimental studies demonstrated that the highest value of the specific cutting resistance $p_{c}=78.14\;N\cdot m^{-1}\;$ occurred during the cutting of eight-layer gauze at a cutting angle α=0° and a cutting speed $V_{c}=0.66\;m\cdot s^{-1}$. Meanwhile, the highest value of the specific cutting work was approximately $L_{jS}=120.00\;J\cdot m^{-2}$ during the cutting of three-layer gauze, also at a cutting speed $V_{c}=0.66\;m\cdot s^{-1}$ for cutting angles α=0°  and α=5°. This study found that $V_{c}=0.66\;m\cdot s^{-1}$ is the threshold cutting speed at which the material is cut. Below this speed, the cutting drum does not have enough momentum to cut the material. Based on the statistical analysis of the obtained test results, it was concluded that there exists a relationship between the independent and dependent variables. The cutting speed has the greatest impact on the parameters of the surgical gauze cutting process. The test results, which have not been found in the worldwide literature to date, constitute a valuable contribution to the development of the theory of surgical gauze cutting. The experimentally determined specific cutting resistance p_c_ and specific cutting work of surgical gauze broaden the knowledge of the materials used in medicine and contribute to the expansion of scientific knowledge in this field.

## 1. Introduction

The value of the global medical device market was USD 512.29 billion in 2022, and forecasts indicate an increase to USD 799.67 billion by 2030, which means a compound annual growth rate (CAGR) of 5.9%. The market value of disposable medical supplies such as gloves, syringes, masks and surgical gauze dressings is a significant part of this market. However, exact data on the value of this segment is not widely available [1].

The dynamic development of this sector is, among other things, related to the increase in the number of surgical procedures, the aging of the population and the growing demands on the quality of medical care.

In Europe, there is a significant number of manufacturers of surgical dressings, such as TZMO, generating revenues in the hundreds of millions of zlotys per year. Even though surgical dressings only make up a small part of the entire medical market, their significance, both on a global and local scale, is considerable, and forecasts indicate further, stable growth.

The market for medical supplies includes traditional products such as bandages, gauze and plasters, as well as modern specialized dressings, including biodegradable and active wound healing materials. Among the key factors driving the development of this market are the growing number of surgical procedures, the increasing incidence of chronic skin conditions resulting from the aging process, the development of new technologies and increased investment in the healthcare sector. Sterile surgical gauze made of cotton or other nonwoven materials is widely used in the dressing of wounds as well as in hygiene and outpatient procedures. They are also used in first aid and for the treatment of heavily oozing or contaminated wounds. These gauzes are loosely woven fabrics with a characteristic nonwoven structure.

Surgical gauze is made with a weaving technique from cotton (*Tela gossypii*), viscose (*Tela cellulosi*) or a mixture of both (*Tela gossypi* and *cellulosi*). The process of weaving consists of interlacing threads at a right angle, while maintaining the appropriate tension of the weft and warp, which ensures the material’s elasticity and strength. Types of gauze are classified according to the number of threads per unit area, the strength of the weft and warp, and the weight. The most commonly used are 13-thread (light) and 17-thread (heavy) gauze [2]. Before surgical gauze can be fully utilized, it has to be cut to specific lengths and packed in sterile paper packaging. This process is carried out by machines equipped with specially designed cutting drums. The specific design and functioning of the drum cutting unit are based on the characteristics of the material being cut—nonwoven fabric of plant origin with a heterogeneous structure and variable physicomechanical properties.

In the scientific literature, only a few publications are available on the topic of cutting surgical gauze. One of the first articles dealing with this topic was published in the world’s largest nursing journal [3], discussing the topic of professional development through the implementation of modern, evidence-based solutions. The issue of cutting dressing materials often occurs in the context of presenting new medical technologies and devices to support the work of medical personnel [4]. One example can be the analysis of cutting nonwoven gauze with specialized scissors within the scope of studies on the Lap-Trainer simulator or references to this process in the context of robotic platforms used in surgery [5].

The analysis of the literature points to numerous studies on the cutting of nonwoven materials, both cotton and synthetic. These works focus on dynamic cutting as a new experimental method [6] and the application of laser cutting in the textile industry [7]. Due to the different reactions of textiles to laser processing, researchers have analyzed the use of the CO_2_ system in optimizing the quality of cut edges [8], as well as the influence of carbonization and oxidation on the variability in nonwoven contours [9].

In the context of the industrial cutting of textiles, the issues of blade wear and its impact on cutting precision are also addressed [10]. One of the studies showed the relationship between cutting force and abrasive wear by analyzing the evolution of roughness and cutting-edge radius. Other studies focused on the impact of energy consumption on cutting multilayer fabrics in the hospitality industry [11] and the logistics of cutting processes [12].

Further analyses focused on energy-efficient cutting presses [13] and on modeling the cutting of polymer-reinforced materials [14], taking into account analytical, numerical and mechanical approaches. The research studies also included the impact of modern, highly absorbent materials on the wear of cutting tools [15].

In the scientific literature, one can also find studies on cutting anisotropic materials, including biological ones. Many studies have focused on modeling the scissor cutting process [16] and analyzing the forces necessary for the precise cutting of soft tissue and bone [17,18]. There are also studies analyzing the impact of cutting force optimization on the speed of patient recovery after surgical procedures [18] and a review of progress in surgical tool design [19]. In the analysis of the literature in the aspect of research on the process of cutting fibrous materials, it should be noted that this topic also concerns materials such as biomass [20,21] and layered materials [22].

Although numerous studies have been conducted on the cutting of various materials, there are no scientific publications on the cutting of surgical gauze using drum cutting units. Furthermore, the small number of studies in this field does not allow for a clear determination of the impact of individual design parameters on the energy consumption of the process. This indicates that the design solutions known so far have been developed mainly on the basis of engineering intuition.

For this reason, the authors of this paper have undertaken to analyze the process of cutting surgical gauze by a drum cutting unit—an issue that is still not fully understood and described.

## 2. Materials and Methods

The issue of cutting surgical gauze is a particularly current topic and is addressed by many authors. Publications in this field mainly concern the determination of the properties of nonwoven fabric subjected to static tensile loads [23,24,25]. There is no information on the quasi-static and dynamic cutting mechanics of the mentioned material.

Therefore, there is an urgent need to supplement knowledge in this area. The authors of this study made an attempt to supplement knowledge in this field through experimental research on the cross-cutting and diagonal cutting of surgical gauze using a special cutting drum design installed on an original test stand, made in-house.

For this reason, the main objectives of this study were to experimentally determine the influence of selected features and parameters of the aforementioned cutting drum design on selected performance characteristics of the surgical gauze cutting process.

The information obtained from the experimental tests will significantly expand the area of knowledge in the field of the machine cutting of surgical gauze. For the purpose of conducting experimental research, a research schedule was planned in which the independent variables were

- Cutting speed $V_{c}$ [$m\cdot s^{-1}$];
- Angle of feeding of surgical gauze α […°];
- Number of layers in the strip of material being cut $h_{w}$ [pc.].

**Figure 1:**
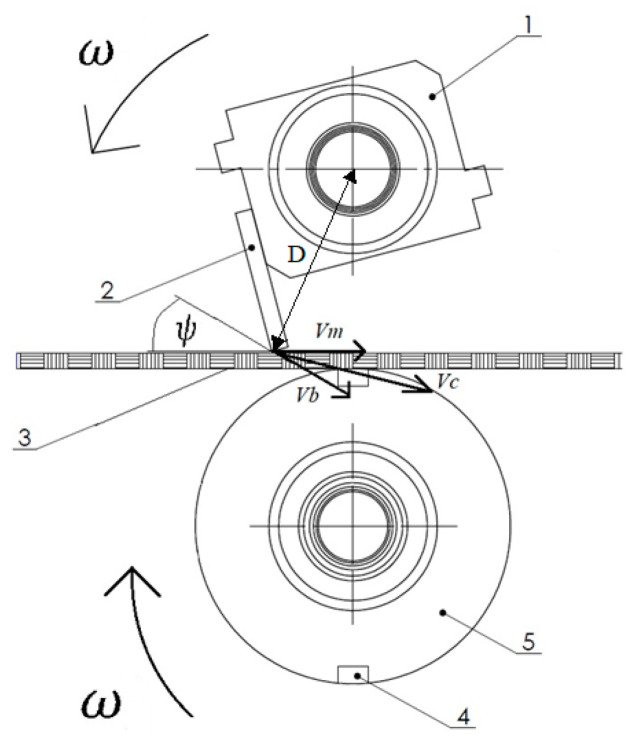
Diagram of cutting surgical gauze: 1—upper cutting drum; 2—cutting knife; 3—surgical gauze strip; 4—counter-cutting strip; 5—lower cutting drum; *ω*—angular speed of the upper and lower cutting drums; ψ—angle between the feeding speed vector $V_{m}$ and the circumferential speed of the drum $V_{b}$.

The cutting speed $V_{c}$ is the speed that the blade reaches when it comes into contact with the material being cut. The cutting speed vector is the resultant of the peripheral speed of the cutting blade $V_{b}$ and the speed at which the material is fed $V_{m}$. The vector of circumferential speed is attached to the circumference of the cutting drum. The linear speed $V_{m}$ is related to the speed of material feeding from the winding shaft, as shown in Figure 1. Therefore, the cutting speed $V_{c}$ was calculated using the formula

(1) $$V_{c}=\sqrt{V_{b}^{2}+V_{m}^{2}+2V_{b}V_{m}cos\psi }.$$

It was assumed that the cutting speed of the drum knife had the following values: 0.66; 0.99; 1.33; 1.66; 2.01; 2.34; 2.67; 3.00; and 3.34 [$m\cdot s^{-1}$]. The specified cutting speeds $V_{c}$ ensure that single and multiple layers of surgical gauze are cut properly, as determined in preliminary studies, and correspond to the cutting speeds achieved in machines for the production of compresses [26].

The angle of feeding of the surgical gauze α (equivalent to the cutting angle of the surgical gauze) is defined as the angle between the center line of the positioning frame of the unwinder and rewinder and the cutting edge of the cutting unit. The values of the cutting angle of surgical gauze were adopted on the basis of the authors’ own studies and studies described in the literature concerning the cross-section of materials of plant origin ($\alpha =0^{0}$) [20]. However, for the purposes of experimental tests, the authors assumed that, apart from being cut transversely ($\alpha =0^{0}$), surgical gauze will also be cut diagonally at an angle (α=5;10 i $15^{0}$). The number of layers $h_{w}$ of cut surgical gauze was defined in terms of strips of cut material consisting of 3, 4 and 8 layers. The value of the number of surgical gauzes $h_{w}$ for the purposes of experimental tests was selected based on the machine’s ability to duplicate gauze. Moreover, such thicknesses correspond to the most commonly available surgical compresses available for general sale [27,28,29,30]. On the other hand, the following were assumed as experience-dependent variables:

- Unit cutting resistance of surgical gauze $p_{c}$ [$N\cdot m^{-1}$];
- Unit work of cutting surgical gauze $L_{jS}$ [$J\cdot m^{-2}$].

The unit cutting resistance of surgical gauze $p_{c}$ is the force N that must be applied to cut a surgical gauze band under specific cutting conditions, and the unit cutting force is related to the length of the cut Δl. Therefore, the unit cutting resistance of surgical gauze $p_{c}$ can be calculated from the equation

(2) $$p_{c}=\frac{N}{\Delta l}$$

However, the unit cutting work $L_{jS}$ is the amount of energy needed E to perform the process of cutting a surgical gauze band, in relation to its cross-sectional area S. Therefore, the unit work of cutting $L_{jS}$ can be calculated from the equation

(3) $$L_{jS}=\frac{E}{S}$$

The experiment was planned according to a 9 × 4 × 3 three-factor cross-classification. During the studies, five replications were used in the sample. The number of replications was determined based on preliminary studies and their statistical evaluation. As the test material intended for cutting, surgical gauze with the properties specified in the raw material documentation was used. Table 1 shows the characteristics of the gauze used for testing.

**Figure 2:**
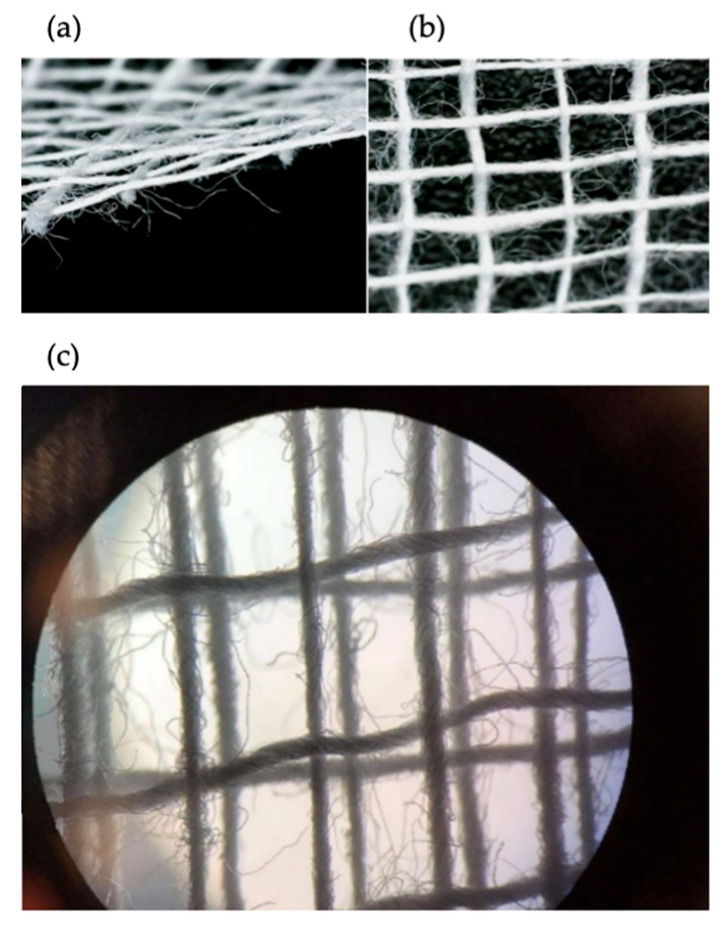
Photos of the gauze used for the tests: (**a**) photo of a cross-section of a single layer of gauze, (**b**) photo of a top view of a single layer of gauze, and (**c**) photo of a 3-layer gauze under a microscope (magnification *p* = 100).

Figure 2 shows photos of surgical gauze intended for testing. The structure of the gauze visible in the photo creates a plain weave. In the image under the microscope, individual cotton fibers and successive layers of gauze are visible. It is worth noting that the layers are not perfectly aligned in a row, but shifted relative to each other.

**Figure 3:**
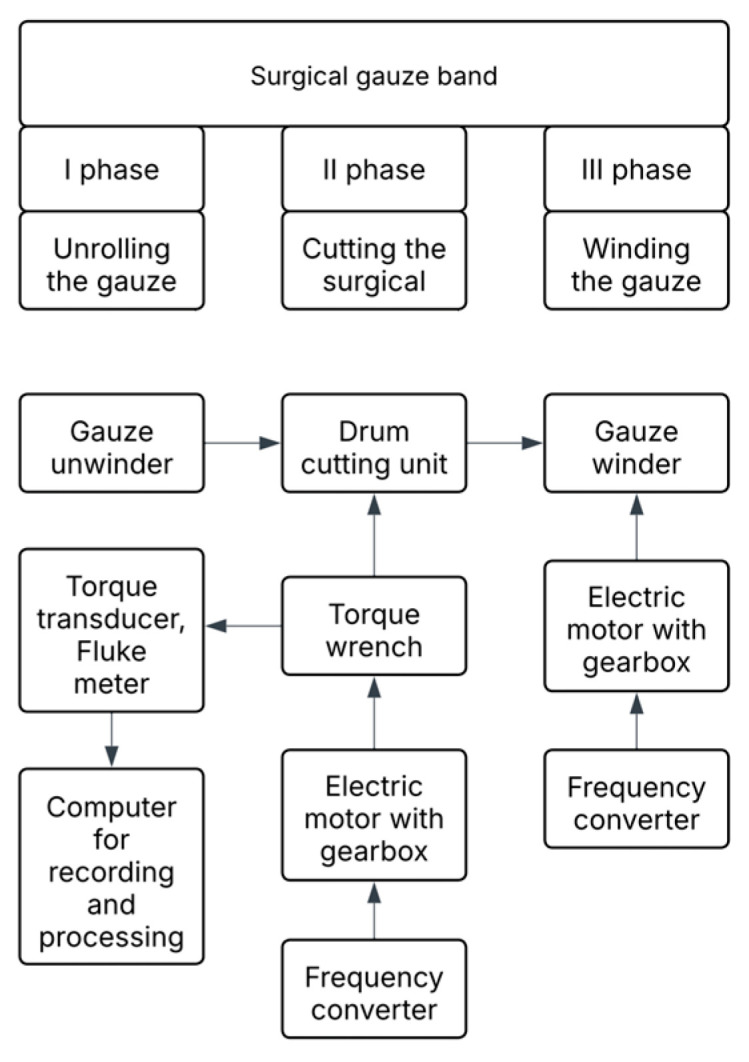
Block diagram of the test stand.

For the purpose of conducting experimental tests, a test stand was designed and constructed to enable the testing of the unit cutting resistance $p_{c}$ and unit cutting work of surgical gauze $L_{jS}.$ The test stand performs the cutting process with a drum cutting unit. The cutting material is fed through a surgical gauze unwinder and rewinder unit. In Figure 3, a block diagram of the test stand is presented.

**Figure 4:**
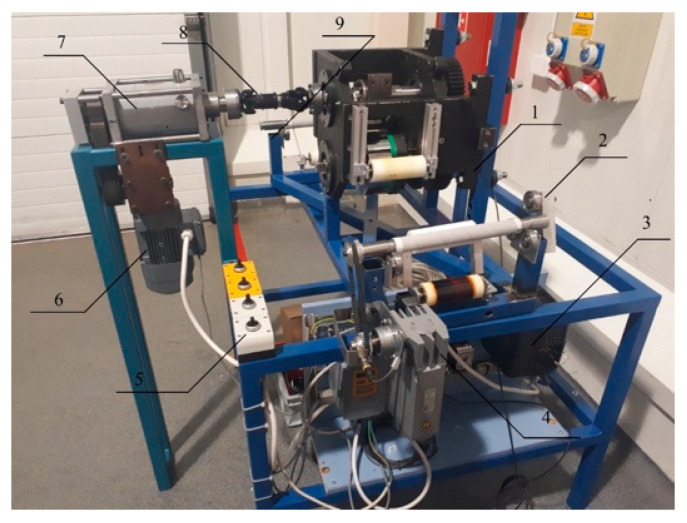
Test stand in view: 1—drum cutting unit; 2—winder unit; 3—control system; 4—electric motor with gearbox for winder; 5—switches to control operation of motors and speed regulation; 6—electric motor with gearbox for cutting unit; 7—torque meter; 8—Cardan coupling; 9—frame of material feeder.

The test stand was designed and constructed according to our own design. It consists of main subassemblies that can be separated into individual modules. Each module has a specific place in the test stand structure and plays an important role in the operation of the entire test stand (Figure 4). The main subassemblies may include

- A construction frame;
- A rotary feeder with a surgical gauze winder and unwinder;
- A drum cutting unit with a straight knife;
- Drives and gears;
- A control system.

**Figure 5:**
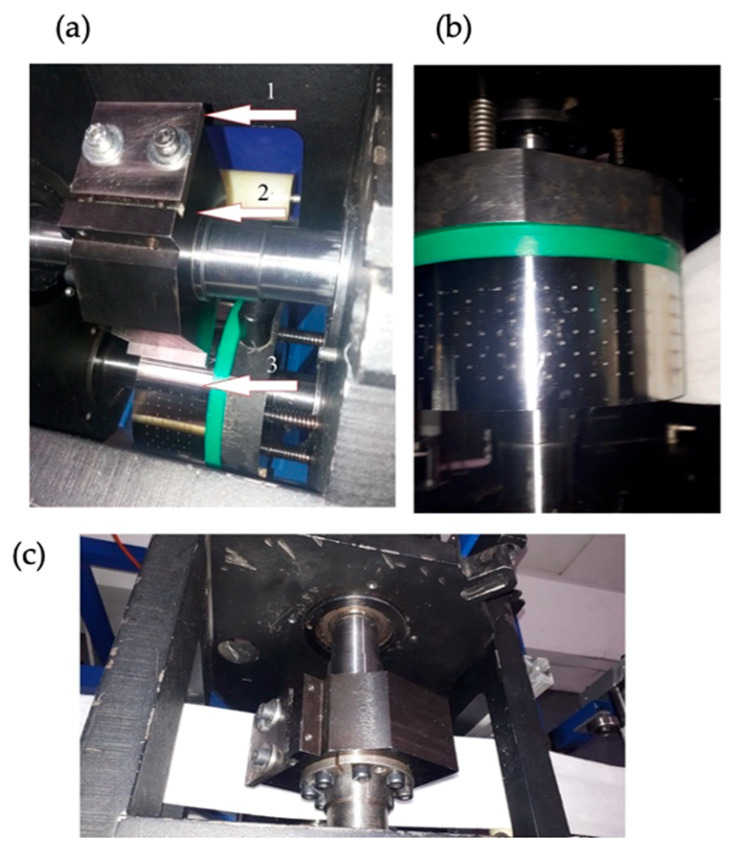
Drum cutting unit used in the test stand: (**a**) inside the cutting unit; (**b**) lower drum; (**c**) upper cutting drum. 1—cutting blade; 2—upper drum; 3—lower drum.

The main unit of the entire test stand is the drum cutting unit that performs the process of cutting surgical gauze. The drum cutting unit is shown in Figure 5. It is equipped with a straight knife embedded in a mounting bracket that is located on the shaft of the upper drum. The blade is made of N5 tool steel with a hardness of 58 HRC. The cutting edge can be sharpened when the blade is worn out. The oblong holes make it possible to adjust the blade in terms of moving it closer to or further away from the counter-cutting edge. The upper shaft of the drum is coupled to the gear shaft of the drive. The rotational speed and torque are transmitted to the upper cutting drum. The drive from the upper drum to the lower drum is transmitted via two gear wheels. The parameters of the wheels are the module m=2 and the number of teeth z = 45.

## 3. Research Methodology

Three-, four- and eight-layer surgical gauzes were used for the studies. The material was stored in a dry room with a humidity of no more than 70% and a temperature equivalent to approximately 20 °C. The main characteristics of the material are shown in Table 2.

**Figure 6:**
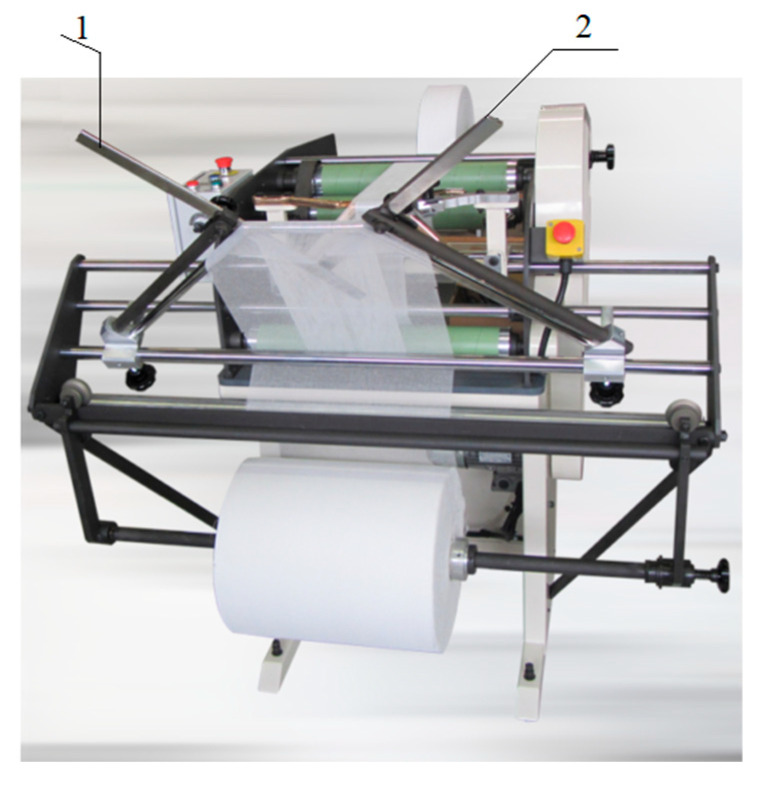
Gauze doubling machine: 1—left folding arm; 2—right folding arm.

The material samples were prepared on a machine used for doubling gauze. The technological process of doubling consists in obtaining a strip composed of several layers of surgical gauze. Surgical gauze composed of several layers of material is obtained during its rewinding. In the process of rewinding the gauze, the special arms of the doubling machine form the direction and plane of the rewinding in such a way that the layers overlap and form a strip composed of several layers. The doubling machine is shown in Figure 6.

The measurements of the idling and operating torque of the drum cutting unit were carried out using an MIR2 torque meter, which was additionally coupled with an MW2006-4 dual-channel meter. This made it possible to measure the torque on the cutting drum shaft and its revolutions.

During the course of the studies, a computer system with data recording software and the self-developed RB01 calculation program were used. In addition, electrical measurements of the power supply for the electric motors driving the drum of the cutting unit and the surgical gauze winder were taken using a Fluke multimeter (Fluke Corporatio, Everett, WA, USA).

Based on the analysis of the occurring idle and operating torques of the drum cutting unit, the average torque needed to overcome the cutting resistance of surgical gauze was obtained from Equation (4):

(4) $${\left(M_{c}\right)}_{s}={\left(M_{b}\right)}_{s}-{\left(M_{j}\right)}_{s},$$

where

${\left(M_{c}\right)}_{s}$—averaged idle torque.

Based on the knowledge of the radius r of the cutting drum and ${\left(M_{c}\right)}_{s}$, it is possible to calculate the circumferential force P attached to the circumference of the cutting drum from the dependence

(5) $$P=\frac{{\left(M_{c}\right)}_{s}}{r}$$

The circumferential force presses the knife edge against the counter-blade and causes it to cut through the surgical gauze. Therefore, the specific cutting resistance $p_{c}$ was calculated from the equation

(6) $$p_{c}=\frac{P}{\Delta l},$$

Length Δl was measured with a millimeter-measuring tape. The unit work of cutting, on the other hand, was determined from the dependence

(7) $$L_{jS}=\frac{2\pi {\left(M_{c}\right)}_{s}}{zhb},$$

where

z—the number of knives (z=1);

h—the height of the material to be cut;

b—the width of the material to be cut.

## 4. Analysis of the Test Results

The results obtained from experimental studies of the surgical gauze cutting process were subjected to statistical analysis, during which the arithmetic means were calculated: $\bar{p_{c}}$ and $\bar{L_{jS}}$ from a given sample for five replications, standard deviations $\sigma_{p_{c}}$ and $\sigma_{L_{jS}}$ and coefficients of variation $\frac{\sigma_{p_{c}}}{p_{c}}$ and $\frac{\sigma_{L_{jS}}}{L_{jS}}$ expressed as a percentage. The conducted regression analysis showed that at the significance level $\alpha_{i}$ = 0.05, with the multiple correlation coefficient R = 0.91, the independent variables $V_{c}$, α and $h_{w}$ have a significant impact on the unit cutting resistance value $p_{c}$.

For the unit cutting resistance of surgical gauze $p_{c}$, the regression function analysis was performed in four stages, rejecting selected coefficients as a result of the significance test performed at the level $\alpha_{i}$ = 0.05.

Finally, the regression equation for the dependent variable $p_{c}$ was accepted in the form of

(8) $$Y_{p_{c}}=-61.48+10.31ln\alpha -133.28lnV_{c}+0.23h_{w}^{2}-0.09\alpha^{2}+60.05V_{c}^{2}-6.37V_{c}^{4}+0.03V_{c}^{8}-4.81\cdot 10^{-7}V_{c}^{16}.$$

**Figure 7:**
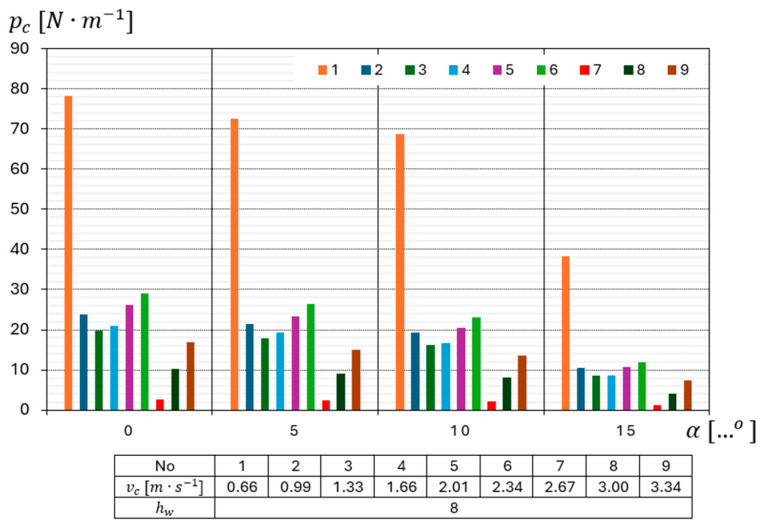
Diagram of the dependence of the unit cutting resistance $p_{c}$ of 8-layer surgical gauze on the cutting angles for all tested cutting speeds $v_{c}$.

The graph in Figure 7 shows the results of the study of the unit cutting resistance of eight-layer surgical gauze $p_{c}$ as a function of cutting angles for all the tested cutting speeds.

The highest value of unit cutting resistance $p_{c}$ = 78.14 $N\cdot m^{-1}$ occurred when cutting was performed at an angle α=0°, while the lowest value of $p_{c}$ was $p_{c}$ = 1.11 $N\cdot m^{-1}$ and occurred when cutting was performed at an angle α=15°.

In the course of the tests, it was found that a cutting speed of $V_{c}$ = 0.66 $m\cdot s^{-1}$ is the limiting cutting speed at which the material is cut through. Below this speed, the cutting drum has too little momentum and insufficient dynamics to cut through the material. In a situation like this, the uncut surgical gauze gets stuck between the upper and lower drums and stops the cutting process. Based on the course of the linear function, it was found that as the cutting speed $V_{c}$ increases, the unit cutting resistance $p_{c}$ decreases. This trend was observed for all measurement series.

**Figure 8:**
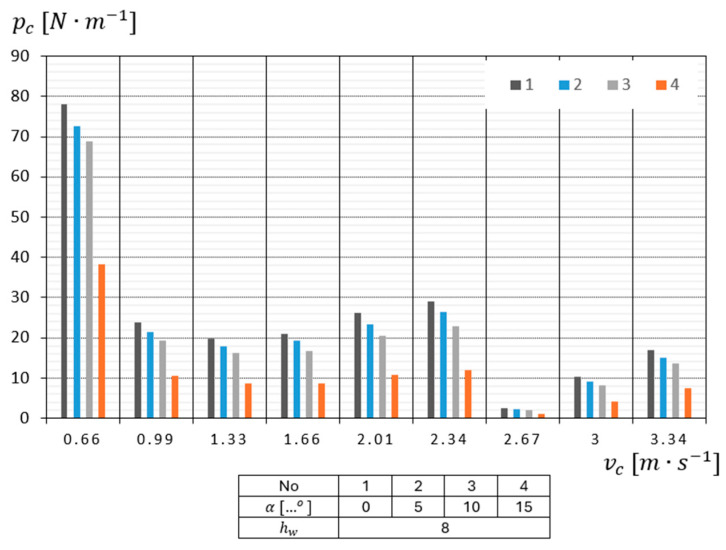
Diagram of the dependence of the unit cutting resistance $p_{c}$ of 8-layer surgical gauze on the cutting speed $v_{c}$ for the four cutting angles tested.

From the analysis of the data in the conducted study, it appears that when cutting eight-layer material, the highest value $p_{c}=78.14\;N\cdot m^{-1}\;$ is obtained for a cutting speed of $V_{c}$ = 0.66 $m\cdot s^{-1}\;$ and a cutting angle of α=0°, while the smallest value $p_{c}=1.11\;N\cdot m^{-1}$ is obtained when cutting the material at a speed of $V_{c}$ = 2.67 $m\cdot s^{-1}$ and at an angle of α=15° (Figure 8).

**Figure 9:**
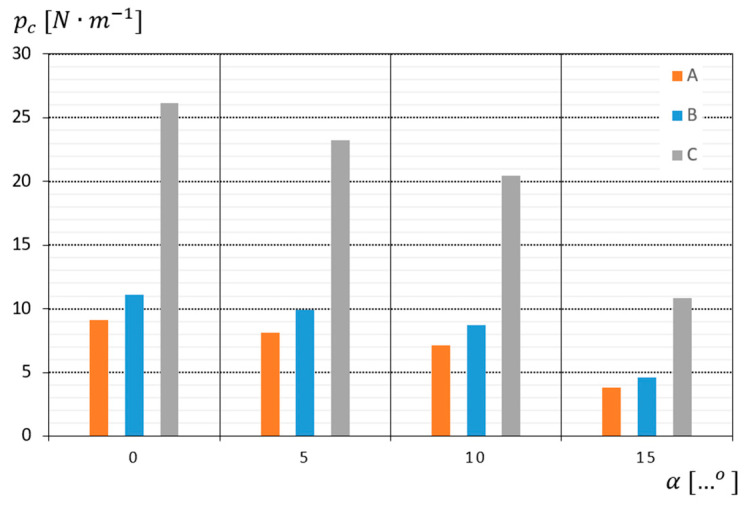
Graph of the dependence of the unit cutting resistance as a function of the cutting angle of the gauze at a speed of $V_{c}$ = 2.01 $m\cdot s^{-1}$ for the following: A—3-layer gauze; B—4-layer gauze; C—8-layer gauze.

On the other hand, when analyzing the impact of the number of layers $h_{w}$ of the cut surgical gauze on its unit cutting resistance $p_{c}$, it should be stated that for all obtained test results within the measuring range of the device, there occurs a dependence where, together with the increase in the number of layers $h_{w}$ of surgical gauze, the unit cutting resistance $p_{c}$ increases. This applies to both transverse α=0° and diagonal cuts at the angles α=0°, 10° and 15° (Figure 9).

From the analysis of the results presented in Figure 9, it can be seen that the highest value $p_{c}=26.12\;N\cdot m^{-1}\;$ was recorded during cross-cutting and the lowest $p_{c}=3.78\;N\cdot m^{-1}\;$ during diagonal cutting at the angle α=15°. The reasons for all the data series observed were that the increase in the unit cutting resistance as a function of the number of layers is similar and amounts to between 18 and 24% between three-layer and four-layer gauze, and 53 and 57% between four-layer and eight-layer gauze.

From the analysis of the obtained test results, it can be clearly stated that increasing the cutting angle results in a reduction in the cutting force, which may indicate an improvement in cutting conditions and a reduction in tool resistance. The most effective angle in terms of cutting force reduction is angle α=15°, while the curvilinear cut (α=0°) results in the greatest resistance.

It can therefore be concluded that the individual cutting resistance depends proportionally on the number of layers of surgical gauze. Similar relationships exist for $V_{c}$ = 1.33, 1.66, 2.0, 2.34, 2.67, 3.00 and 3.34 $m\cdot s^{-1}$, wherein the unit cutting resistance $p_{c}$ decreases approximately proportionally for transverse cutting α=0° and at the angles α=5°, 10° and 15°.

For the unit work of cutting surgical gauze $L_{jS}$, the regression function analysis was performed in three stages, rejecting the selected coefficients as a result of the conducted significance test at the level $\alpha_{i}$ = 0.05. Finally, the regression equation for the dependent variable was adopted in the form of

(9) $$Y_{L_{jS}}=-268.051+535.26V_{c}-6.56lnh_{w}-262.12lnV_{c}-157.56V_{c}^{2}+7.43V_{c}^{4}-0.02\alpha^{8}+3.11\cdot 10^{-7}V_{c}^{8}-2.19\cdot 10^{-8}h_{w}^{16}$$

The conducted regression analysis showed that at the significance level $\alpha_{i}$ = 0.05, with the multiple correlation coefficient R = 0.98, the independent variables $V_{c}$, α and $h_{w}$ have a significant impact on the value of a unit of cutting work $L_{jS}$.

**Figure 10:**
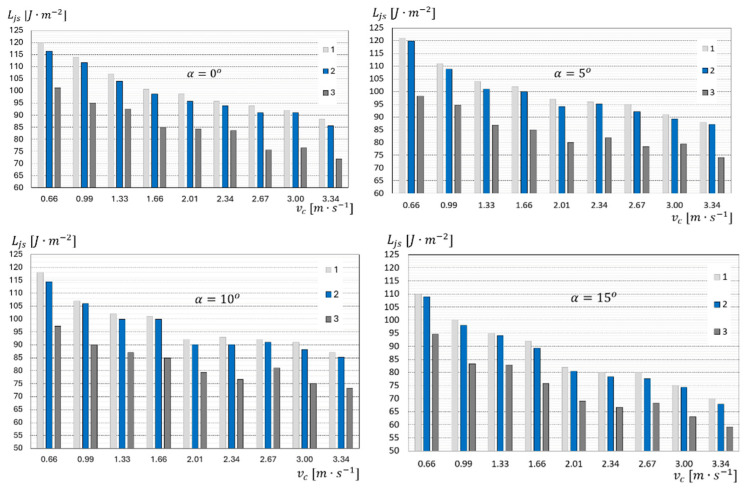
Graph of the unit cutting work of surgical gauze $L_{js}$ as a function of cutting speed $v_{c}$ for cutting angles α=0°,5°, 10° and 15°, for the following: 1—three-layer gauze, $h_{w}=3$; 2—four-layer gauze, $h_{w}=4$; 3—eight-layer gauze, $h_{w}=8$.

The diagrams presented in Figure 10 refer to four feeding angles α and all the types of surgical gauze that were subject to cutting. According to the diagram, together with the increase in cutting speed $V_{c}$, the unit cutting work $L_{jS}$ decreases for all the tested types of material. The more layers $h_{w}$ of the material there are to be cut, the lower the value of $L_{js}$ is. Such a correlation is evident for all the cutting speed $V_{c}$ options.

The highest value $L_{js}$ = 120.00 $J\cdot m^{-2}$ occurred for the cutting speed $V_{c}$ = 0.66 $m\cdot s^{-1}$ and the number of material layers $h_{w}$ = 3. The lowest value $L_{js}$ = 59.07 $J\cdot m^{-2}$ was obtained for the cutting speed $V_{c}$ = 3.34 $m\cdot s^{-1}$ and the material thickness $h_{w}$ = 8. It was observed that for cutting eight-layer material, on average, 15 $J\cdot m^{-2}$ less energy was used than for cutting material $h_{w}$ with three and four layers. This means that as more layers of material are added, the unit cutting work $L_{js}$ decreases. This is an important consideration in terms of the energy consumption of the process. A certain minimum level of cutting work $L_{jS}$ is consumed by the idling of the drum cutting unit. The work required to start the drum cutting unit and to accelerate the drums is relatively high compared to the work required to cut a single layer of surgical gauze. Therefore, the highest unit cutting work occurs during start-up and at low rotational speeds of the cutting drum. On the basis of Figure 10, it can be concluded that within the cutting speed range from $V_{c}$ = 0.66 to 2.34 $m\cdot s^{-1}$ j, the unit cutting work of four- and eight-layer gauze had a lower value than in the three-layer gauze cutting process. Above a speed of $V_{c}$ = 2.34 $m\cdot s^{-1}$, the unit cutting work $L_{jS}$ has a varied course and the trend of changes cannot be clearly determined.

## 5. Conclusions

1. The conducted experimental studies of the surgical gauze cutting process clearly showed that an important impact on the unit cutting resistance $p_{c}$ and unit cutting work $L_{jS}$ is exerted by all the independent variables, i.e., cutting speed $V_{c}$, gauze feed angle α and the number of layers $h_{w}$ in the strip of material to be cut.
2. The highest value of unit cutting resistance $p_{c}=78.14$ $N\cdot m^{-1}$ for the variable configurations determined according to the test program occurred at a cutting speed of $V_{c}$ = 0.66 $m\cdot s^{-1}$. During the course of the tests, it was noted that this was the limiting speed below which the drum cutting unit was not able to cut the gauze.
3. The unit cutting resistance is significantly reduced once the minimum cutting speed of $V_{c}$ = 0.66 $m\cdot s^{-1}$ is exceeded and does not reach such high values for the remaining independent variable configurations during the conducted experiment.
4. The thickness of the material in the strip has a significant impact on the cutting resistances of surgical gauze.
5. The unit cutting resistance was measured to be more than twice as high for a gauze with the number of layers $h_{w}$ = 8. This relationship exists for all the cutting speeds $V_{c}.$ When comparing the results of the tests of the unit cutting resistance $p_{c}$ of an eight-layer gauze to a four-layer gauze, it was noticed that the unit cutting resistance $p_{c}$ was half as high for a gauze with the number of layers $h_{w}$ = 8.
6. It was observed that within the range of cutting speeds $V_{c}$ = 0.66–3.34 $ms^{-1}$ and material feed angles α = 0–15°, the unit cutting resistance $p_{c}$ of surgical gauze decreases. Based on the research, it was concluded that the cutting speed has a greater impact on the unit cutting resistance $p_{c}$ than the material feed angle α.
7. Together with the increase in cutting speed $V_{c}$ from 0.66 to 3.34 $m\cdot s^{-1}$ and the material feed angle α from 0° to 15°, the unit cutting work $L_{js}$ was reduced. Based on the analysis of the test results, it was concluded that a greater impact on the unit cutting work value $L_{js}$ is exerted by the cutting speed $V_{c}$ rather than by the material feed angle α (the same trend occurs in the case of the unit $p_{c}$).

## Figures and Tables

**Table 1:** Characteristics of the surgical gauze used for the tests.

Parameter	Unit	Testing Method	Type of Gauze
			13 Threads/cm^2^
number of threads in the warp	pcs./100 mm	PN-EN 14079 [31]	70 ± 4
number of threads in the weft	pcs./100 mm		60 ± 4
surface weight	$g\cdot m^{-2}$		≥17
breaking strength in the warp	N/50 ${mm}^{-1}$		≥35
breaking strength of the weft	N/50 ${mm}^{-1}$		≥20
length of a roll of gauze	lin.m.	-	≥2500 ± 10

**Table 2:** Material and geometric properties of the surgical gauze samples for cutting.

Sample Characteristics	A	B	C
Average sample length $\left[m\right]$	100		
Sample thickness $\left[mm\right]$	2.00	2.50	5.00
Number of layers	3	4	8
Sample width $\left[cm\right]$	9–9.5		
Sample humidity $\left[\%\right]$	6		

## Data Availability

All the computational data from mathematical simulations are included in the article.
